# New Prediction Models of Functional Outcome in Acute Intracerebral Hemorrhage: The dICH Score and uICH Score

**DOI:** 10.3389/fneur.2021.655800

**Published:** 2021-05-05

**Authors:** Wen-Song Yang, Yi-Qing Shen, Xiao Wei, Li-Bo Zhao, Qing-Jun Liu, Xiong-Fei Xie, Zhi-Wei Zhang, Lan Deng, Xin-Ni Lv, Shu-Qiang Zhang, Xin-Hui Li, Qi Li, Peng Xie

**Affiliations:** ^1^Department of Neurology, The First Affiliated Hospital of Chongqing Medical University, Chongqing, China; ^2^National Health Commission Key Laboratory of Diagnosis and Treatment on Brain Functional Diseases, The First Affiliated Hospital of Chongqing Medical University, Chongqing, China; ^3^Department of Traditional Chinese Medicine, Chongqing Medical and Pharmaceutical College, Chongqing, China; ^4^Department of Neurology, Yongchuan Hospital of Chongqing Medical University, Chongqing, China; ^5^Chongqing Key Laboratory of Cerebrovascular Disease Research, Yongchuan Hospital of Chongqing Medical University, Chongqing, China; ^6^Department of Radiology, The First Affiliated Hospital of Chongqing Medical University, Chongqing, China

**Keywords:** intracerebral hemorrhage, ICH score, hematoma expansion, outcome, NCCT marker, computed tomography

## Abstract

**Objectives:** The original intracerebral hemorrhage (oICH) score is the severity score most commonly used in clinical intracerebral hemorrhage (ICH) research but may be influenced by hematoma expansion or intraventricular hemorrhage (IVH) growth in acute ICH. Here, we aimed to develop new clinical scores to improve the prediction of functional outcomes in patients with ICH.

**Methods:** Patients admitted to the First Affiliated Hospital of Chongqing Medical University with primary ICH were prospectively enrolled in this study. Hematoma volume was measured using a semiautomated, computer-assisted technique. The dynamic ICH (dICH) score was developed by incorporating hematoma expansion and IVH growth into the oICH score. The ultra-early ICH (uICH) score was developed by adding the independent non-contrast CT markers to the oICH score. Receiver operating characteristic curve analysis was used to compare performance among the oICH score, dICH score, and uICH score.

**Results:** There were 310 patients in this study which included 72 patients (23.2%) with hematoma expansion and 58 patients (18.7%) with IVH growth. Of 31 patients with two or more non-contrast computed tomography markers, 61.3% died, and 96.8% had poor outcomes at 90 days. After adjustment for potential confounding variables, we found that age, baseline Glasgow Coma Scale score, presence of IVH on initial CT, baseline ICH volume, infratentorial hemorrhage, hematoma expansion, IVH growth, blend sign, black hole sign, and island sign could independently predict poor outcomes in multivariate analysis. In comparison with the oICH score, the dICH score and uICH score exhibited better performance in the prediction of poor functional outcomes.

**Conclusions:** The dICH score and uICH score were useful clinical assessment tools that could be used for risk stratification concerning functional outcomes and provide guidance in clinical decision-making in acute ICH.

## Introduction

Intracerebral hemorrhage (ICH) is the second most common stroke subtype, high in mortality and morbidity (1). The case-fatality rate of patients with ICH ranges from 35 to 59%, and more than 60% of survivors have a poor prognosis (2). In previous studies, independent predictors of poor functional outcomes included age, hematoma volume, intraventricular hemorrhage (IVH), Glasgow Coma Scale (GCS) score, and infratentorial hemorrhage (3, 4). Hemphill et al. (5, 6) integrated these variables to establish the original ICH (oICH) score, which has been a severity score most commonly used in clinical ICH research.

However, acute ICH is a dynamic process, such that ~30% of patients will experience early hematoma expansion (7, 8). IVH growth has been reported in ~20% of patients with ICH (9). Recent studies suggested that both early hematoma expansion and IVH growth were independently associated with poor outcomes (7–10). The predictive accuracy of the oICH score may be influenced by hematoma expansion or IVH growth in acute ICH. To ensure optimal patient care, clinicians require accurate information to perform effective risk stratification concerning patient outcomes.

Schneider et al. (11) provided a prognostic score by adding the computed tomography angiography (CTA) spot sign to the oICH score to predict poor outcomes in ICH patients. However, identifying the spot sign requires a CTA examination, which is not readily available in most hospitals during the acute phase of ICH. Non-contrast computed tomography (NCCT) is the most widely used examination for the evaluation of ICH in ultra-early ICH (uICH). Several NCCT markers have emerged as promising indicators of hematoma expansion and poor outcomes (12). Notably, NCCT markers could serve as alternatives to the CTA spot sign for predicting hematoma expansion if CTA examination is unavailable (13–16). Thus, the NCCT markers might improve the ability of the oICH score to predict clinical, functional outcomes in patients with acute ICH.

This study was performed to investigate the prognostic value of the oICH score with respect to functional outcomes in patients with acute ICH. Moreover, it aimed to determine whether the inclusion of the NCCT markers into the oICH score could accurately stratify acute ICH patients in terms of 90-day functional outcomes.

## Materials and Methods

### Study Design and Patient Selection

Patients admitted to the First Affiliated Hospital of Chongqing Medical University with primary ICH between January 2012 and July 2017 were retrospectively analyzed in this research. In total, 310 patients with primary ICH were included in the analysis (Supplementary Figure 1). Patients with ICH were included if they had an initial NCCT scan performed within 6 h after ICH onset and a follow-up NCCT scan within 36 h after the initial NCCT scan. All research procedures and protocols were performed following the ethical standards of the Declaration of Helsinki.

### Clinical Data Collection and Image Analysis

Baseline demographics, medical comorbidities, clinical admission status, laboratory data, and imaging data were recorded. We evaluated the modified Rankin Scale (mRS) score at 3 months by trained staff. All images were retrospectively assessed by two experienced reviewers who were blinded to clinical characteristics and patient outcomes. The discrepancy between the two reviewers was settled through joint discussion until a consensus was reached. All NCCT markers were judged according to the previous definition.

Primary ICH was classified according to hematoma location as deep, lobar, or infratentorial hemorrhage. The parenchymal hematoma volumes and IVH volumes were measured using semiautomated, computer-assisted volumetric software (Mimics Software, version 20.0; Materialise NV, Leuven, Belgium). Briefly, a mask with predefined values of 44–99 Hounsfield units was constructed for the region of interest. The hematoma segmentation accuracy was confirmed by region growing with manual inspection. The connected hematoma in both parenchymal and ventricular aspects was manually segmented (Supplementary Figure 2). Hematoma volumes were calculated by the software and verified by trained staff.

Blend sign, black hole sign, CT hypodensities, and island sign were defined as previously described (17–20). Significant hematoma expansion was defined as an increase exceeding 33% or 6 ml from the baseline parenchymal hematoma volume (8). IVH growth was defined as either a delayed IVH on follow-up CT or an absolute growth of IVH volume >1 ml from baseline CT to follow-up CT (9, 10). Poor outcomes were defined as an mRS score of 4–6 at 90 days, as described previously (21, 22). We developed the dynamic ICH (dICH) score by incorporating hematoma expansion and IVH growth into the oICH score and further established the uICH score by adding the independent NCCT markers to the oICH score.

### Statistical Analyses

We performed all statistical analyses using SPSS 21.0 (SPSS, Chicago, IL, USA) and MedCalc version 11.4.2. The receiver operating characteristic curves were drawn to calculate the area under the curve (AUC), sensitivity, and specificity. We further compared the performance of the oICH score, the dICH score, and the uICH score for predicting 30-day mortality, 90-day mortality, and poor outcome using the approach described by DeLong et al. (23). The chi-squared test, Fisher's exact test, Student's *t*-test, or Mann–Whitney *U*-test were performed as appropriate. Multivariate logistic regression analysis was used to select independent factors associated with poor outcomes by including all variables with *P* ≤ 0.1 in the univariate analysis. The dICH score and the uICH score were developed using the results of the multivariate logistic regression analysis. The level of significance was set at a *P*-value of < 0.05.

## Results

### Study Population and Clinical Characteristics

In the study, the mean patient age was 59.3 ± 12.3 years. The median time from symptom onset to baseline CT scan was 2 h (interquartile range, 1–4 h). There were 47 patients (15.2%) with blend sign, 44 patients (14.2%) with black hole sign, 100 patients (32.3%) with CT hypodensities, and 46 patients (14.8%) with island sign. Interobserver agreement were excellent for evaluation of blend sign [κ = 0.80 (95% confidence interval; 0.70–0.89)], black hole sign [κ = 0.87 (95% confidence interval; 0.78–0.95)], CT hypodensities [κ = 0.85 (95% confidence interval; 0.78–0.91)], and island sign [κ = 0.89 (95% confidence interval; 0.80–0.96)].

Of the 310 patients, 72 (23.2%) had hematoma expansion, and 58 (18.7%) had IVH growth. Patients with poor outcomes had older age (*P* = 0.002), lower admission GCS score (*P* < 0.001), higher baseline IVH volume (*P* < 0.001), larger baseline hematoma volume (*P* < 0.001), more frequent IVH on initial CT (*P* < 0.001), more frequent hematoma expansion (*P* < 0.001), and more frequent IVH growth (*P* < 0.001; Table 1). Furthermore, patients with poor outcomes were more likely to have the presence of blend sign, black hole sign, CT hypodensities, and island sign (all *P* < 0.001; Table 1). The oICH score, dICH score, and uICH score were significantly higher in patients with poor outcomes than those without, respectively (all *P* < 0.001; Table 1).

**Table 1:** Comparison of demographic, clinical, and imaging characteristics and outcome between patients with and without poor outcome.

**Variables**	**Poor outcome (*n* = 120, 38.7%)**	**Good outcome (*n* = 190, 61.3%)**	***P*-value**
**Demographic**			
Mean age, year (SD)	62.0 (13.2)	57.6 (11.5)	**0.002**
Sex, male, *n* (%)	82 (68.3)	117 (61.6)	0.227
**Clinical characteristics**			
Alcohol consumption, *n* (%)	50 (41.7)	78 (41.1)	0.915
Smoking, *n* (%)	59 (49.2)	81 (42.6)	0.260
Diabetes mellitus, *n* (%)	16 (13.3)	18 (9.5)	0.289
History of hypertension, *n* (%)	89 (74.2)	130 (68.4)	0.279
Admission SBP, mmHg (SD)	174.9 (32.8)	168.9 (24.6)	**0.089**
Admission DBP, mmHg (SD)	100.1 (22.4)	98.6 (14.8)	0.517
Admission GCS score, median (IQR)	11 [7–14]	14 [13–15]	** <0.001**
**Imaging features**			
Time from onset to CT, h (IQR)	2 [1–3]	2 [1–4]	0.292
Presence of IVH on initial CT, *n* (%)	57 (47.5)	39 (20.5)	** <0.001**
Baseline ICH volume, mL (IQR)	19.2 [11.1–31.0]	10.7 [6.3–16.6]	** <0.001**
Hematoma expansion, *n* (%)	51 (42.5)	21 (11.1)	** <0.001**
Baseline IVH volume, mL (IQR)	0 [0–7.1]	0 [0–0]	** <0.001**
IVH growth, *n* (%)	51 (42.5)	7 (3.7)	** <0.001**
Blend sign, *n* (%)	29 (24.2)	18 (9.5)	** <0.001**
Black hole sign, *n* (%)	37 (30.8)	7 (3.7)	** <0.001**
Island sign, *n* (%)	38 (31.7)	8 (4.2)	** <0.001**
CT hypodensities, *n* (%)	60 (50.0)	40 (21.1)	** <0.001**
**ICH Locations**			
Deep hemorrhage, *n* (%)	95 (79.2)	155 (81.6)	0.601
Lobar hemorrhage, *n* (%)	15 (12.5)	23 (12.1)	0.918
Infratentorial hemorrhage, *n* (%)	10 (8.3)	13 (6.8)	0.626
**ICH scores**			
oICH score, median (IQR)	2 [1–2]	0 [0–1]	** <0.001**
uICH score, median (IQR)	2 [1–3]	1 [0–1]	** <0.001**
dICH score, median (IQR)	2 [1.25–3.75]	0 [0–1]	** <0.001**

*ICH, intracerebral hemorrhage; CT, computed tomography; GCS, Glasgow Coma Scale; IVH, intraventricular hemorrhage; IQR, interquartile range; SD, standard deviation; mRS, modified Rankin scale; SBP, systolic blood pressure; DBP, diastolic blood pressure; oICH, original intracerebral hemorrhage; dICH, dynamic intracerebral hemorrhage; uICH, ultra-early intracerebral hemorrhage*.

### Multivariate Logistic Analysis and Establishment of New Scores

After adjustment for the potential confounders, the following factors could independently predict poor outcomes in the multivariate logistic regression analysis: age, baseline GCS score, presence of IVH on initial CT, baseline ICH volume, hematoma expansion, IVH growth, blend sign, black hole sign, and island sign (Table 2). The dICH score and uICH score were developed from Model 1 and Model 2, respectively. Each independent factor was assigned points based on its strength of association with the functional outcomes, as shown in Table 3.

**Table 2:** Multivariate analysis of predictors for poor outcome.

**Variable**	**Odds ratio**	**95% confidence interval**	***P*-value**
**Model 1** ^**a**^			
Age, year^*^	1.04	1.01–1.07	**0.008**
Admission GCS score^*^	0.76	0.68–0.85	** <0.001**
Presence of IVH on initial CT	3.76	1.46–9.67	**0.006**
Baseline ICH volume, ml^*^	1.08	1.04–1.11	** <0.001**
Hematoma expansion	2.81	1.14–6.93	**0.025**
IVH growth	5.39	1.84–15.77	**0.002**
**Model 2** ^**b**^			
Age, year^*^	1.04	1.01–1.07	**0.004**
Admission GCS score^*^	0.76	0.68–0.85	** <0.001**
Presence of IVH on initial CT	8.89	3.68–21.46	** <0.001**
Baseline ICH volume, ml^*^	1.06	1.02–1.09	**0.002**
Blend sign	4.70	1.88–11.77	**0.001**
Black hole sign	4.17	1.19–14.57	**0.025**
Island sign	5.50	1.82–16.60	**0.002**

*ICH, intracerebral hemorrhage; CT, computed tomography; GCS, Glasgow Coma Scale; IVH, intraventricular hemorrhage; SBP Systolic blood pressure; oICH, original intracerebral hemorrhage; dICH, dynamic intracerebral hemorrhage; uICH, ultra-early intracerebral hemorrhage.*

* *Per unit change in regressor.*

a *Adjusted for admission systolic blood pressure and baseline IVH volume.*

b *Adjusted for admission systolic blood pressure, baseline IVH volume, and CT hypodensities*.

**Table 3:** Determinants of the oICH score, dICH score, and uICH score.

**Component**	**Points**		
	**oICH score**	**dICH score**	**uICH score**
**Admission GCS score**			
3–4	2	2	2
5–12	1	1	1
13–15	0	0	0
**Baseline ICH volume, mL**			
≥30	1	1	1
<30	0	0	0
**Presence of IVH**			
Yes	1	1	1
No	0	0	0
**Infratentorial hemorrhage**			
Yes	1	1	1
No	0	0	0
**Age, years**			
≥80	1	1	1
<80	0	0	0
**Hematoma expansion**	Not mentioned		Not mentioned
Yes		1	
No		0	
**IVH growth**	Not mentioned		Not mentioned
Yes		1	
No		0	
**Blend sign**	Not mentioned	Not mentioned	
Yes			1
No			0
**Black hole sign**	Not mentioned	Not mentioned	
Yes			1
No			0
**Island sign**	Not mentioned	Not mentioned	
Yes			1
No			0
**Total Score**	0–6	0–8	0–9

*GCS, Glasgow Coma Scale; ICH, intracerebral hemorrhage; IVH, intraventricular hemorrhage; oICH, original intracerebral hemorrhage; dICH, dynamic intracerebral hemorrhage; uICH, ultra-early intracerebral hemorrhage*.

### Comparison of the Intracerebral Hemorrhage Scores

**Figure 1:**
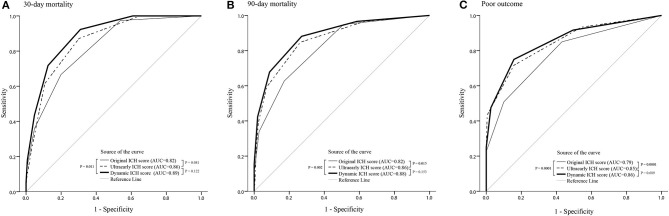
ROC curves of the oICH score, dICH score, and uICH score for prediction of 30-day mortality **(A)**, 90-day mortality **(B)**, and poor outcome **(C)**, respectively. ICH, intracerebral hemorrhage; ROC, receiver operating characteristic; oICH, original intracerebral hemorrhage; dICH, dynamic intracerebral hemorrhage; uICH, ultra-early intracerebral hemorrhage.

**Figure 2:**
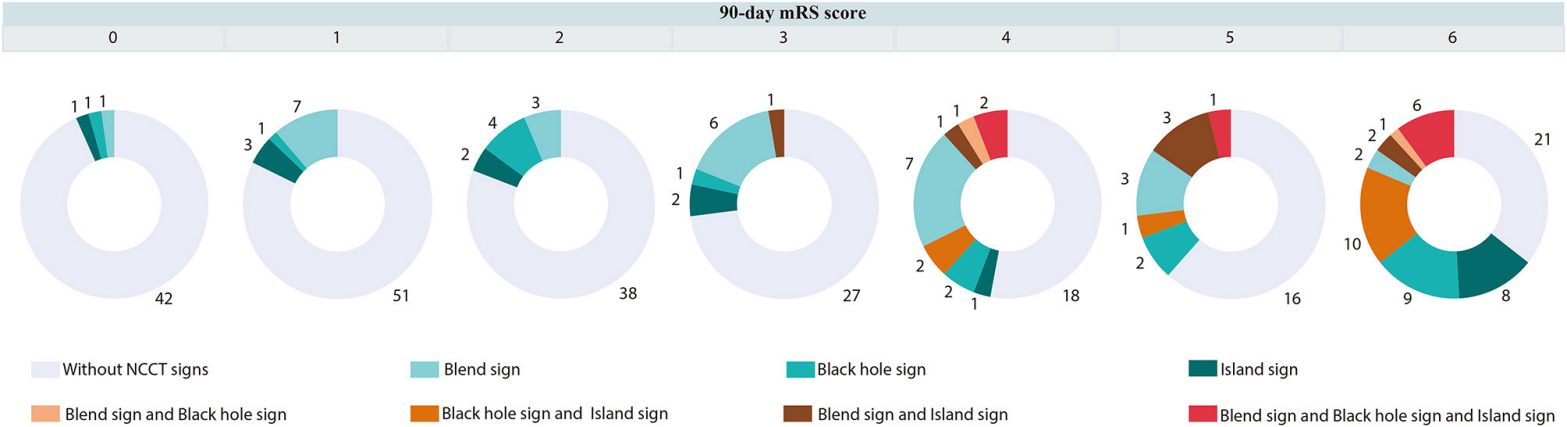
Distribution of mRS in patients with or without blend sign, and (or) black hole sign, and (or) island sign. mRS indicates a modified Rankin Scale.

The accuracies of the oICH score, dICH score, and uICH score for predicting 30-day mortality, 90-day mortality, and poor outcomes were illustrated in Supplementary Table 1. In comparison with the oICH score, the dICH score exhibited better performance in predicting 30-day mortality, 90-day mortality, and poor outcome, respectively (all *P* < 0.05; Figure 1). The uICH score has a higher performance in predicting 90-day mortality and poor outcome compared with the oICH score, respectively (both *P* < 0.05; Figure 1). There were no significant differences between the dICH score and uICH score in predicting 30-day mortality, 90-day mortality, and poor outcome, respectively (Figure 1). Notably, there were 31 patients with two or more NCCT markers. Of these 31 patients, 61.3% died within 90 days of follow-up, and 96.8% had poor outcomes of mRS 4–6 (Figure 2).

## Discussion

This study evaluated the clinical utility of the dICH score and uICH score in the context of acute ICH. Our findings suggested that the performances of the dICH score and uICH score were better than the oICH score in predicting 90-day mortality and poor outcomes in patients with acute ICH.

The prediction of functional outcomes is important for aiding clinicians in clinical decision-making concerning patients with ICH. The oICH score has good predictive ability in terms of risk stratification to predict poor outcomes and is now the standardized score for assessment of patients with spontaneous ICH (5, 6). Although many ICH severity scores have been derived thus far, there is no emerging standard superior to the oICH score (24). This is presumably because the oICH score comprises several core prognostic variables (i.e., age, hematoma volume, IVH, and ICH location) and a severity scale (GCS score).

Hematoma growth is relatively common in patients presented within 24 h after ICH onset; it is especially common in those who present within 6 h. Notably, 50–82% of hematoma growth has been reported to occur within 6 h after ICH onset; this decreases to 17–19% between 6 and 24 h (25, 26). Furthermore, hematoma expansion is the main cause of early neurologic deterioration with a reduction in GCS score (14, 27). Dynamic changes in IVH volume have also been shown to independently predict poor outcomes in ICH patients (10, 11). Importantly, the subtypes of IVH growth (including delayed IVH and IVH volume expansion) are considered independent predictors of poor outcomes (28–30). Thus, the elements of the oICH score, such as hematoma volume and GCS score, may change in the presence of active bleeding, and the ability of the oICH score to predict outcomes may be reduced. Following the incorporation of hematoma expansion into the oICH score, the dICH score showed significantly better performance in the prediction of clinical, functional outcomes compared with the oICH score.

Recently, Sembill et al. (31) established the max-ICH score, which included National Institutes of Health Stroke Scale (NIHSS) score, age, intraventricular hemorrhage, oral anticoagulation, lobar ICH volume ≥ 30 ml, and non-lobar ICH volume ≥ 10 ml, and made a more detailed classification of the variable of age and NIHSS score. The max-ICH score had higher accuracy in the assessment of functional outcomes when compared with the oICH score. However, in subsequent external validation of the max-ICH score, Schmidt et al. (24) found that the prognostic values did not significantly differ between the max-ICH score and oICH score significant difference. Our findings imply that the differences between the prior two studies were related to differences in the course of ICH among patients (e.g., from symptom onset to ICH score assessment). Measurements of the GCS score, the NIHSS score, hematoma volume, and IVH status were performed at an early stage (<24 h) in the study by Sembill et al. (31), whereas they were performed on day 5 in the study by Schmidt et al. (24). Furthermore, Lun et al. (32) found that the oICH score using 24-h imaging had better prognostic accuracy in predicting 3 months mortality compared with an examination at the time of initial presentation. Taken together, these findings indicate the admission data in the acute phase may limit the predictive performance of the oICH score. The dICH score could aid in more accurate risk stratification. Thus, the frequent clinical evaluation and follow-up imaging examination in patients with acute ICH are important for assisting in clinical decision-making.

Notably, many ICH patients arrive at the emergency department within several hours due to the rapid symptom onset. In addition, a recent secondary analysis of the ATACH2 trial results suggested that ultra-early blood pressure reduction was associated with a reduced rate of hematoma growth and improved functional outcomes in ICH patients (33). Therefore, outcome prediction within a few hours after ICH onset is important for clinicians to delineate the potential benefits of aggressive care. In the PREDICT cohort, a prognostic score was defined by incorporating the spot sign into the oICH score, but the spot sign ICH score did not alter the accuracy for predicting poor outcomes compared with the oICH score (11). Although numbers of spot signs have been associated with risk stratification concerning hematoma expansion (34, 35), the relationship between numbers of spot signs and poor outcomes remains unclear. Therefore, the accuracy of the oICH score in predicting poor outcomes of ICH may not be improved by increasing the number of spot signs.

Previous studies suggested that blend sign, black hole sign, hypodensities, and island sign were independently associated with poor outcome in patients with ICH (20, 36–39). Conversely, patients with benign ICH who did not have those imaging markers were less likely to experience hematoma growth and were reported to have good functional outcomes. The model of expansion-prone hematoma composed of blend sign, black hole sign, and island sign has higher performance for predicting poor outcomes than any single NCCT sign (40). Furthermore, it is interesting to note that when two or more NCCT markers are present in a hematoma, the probability of a poor prognosis is greatly increased. Of 31 patients with two or more NCCT markers, 61.3% died, and 96.8% had poor outcomes at 90 days. Thus, after the addition of the blend sign, black hole sign, and island sign to the oICH score, the uICH score showed meaningful improvement with respect to the accuracy of outcome prediction in the uICH when compared with the oICH score. Importantly, we found that the diagnostic performances of the dICH score and the uICH score were similar for predicting 30-day mortality, 90-day mortality, and poor outcomes, respectively. In the present study, the dICH score showed a larger AUC than the oICH score. However, a notable strength of the uICH score is that it can be calculated during the initial evaluation of ICH patients. We note that the AUC of the dICH score was greater than that of the uICH score, but it is reasonable because the dICH score included information concerning hematoma expansion and IVH growth to the uICH score. Moreover, the uICH score used information from the initial evaluation and had a predictive capacity similar to that of the dICH score. The uICH score might be considered superior to the dICH score because the uICH score can be calculated on admission and has prognostic information similar to that of the dICH score. The uICH score may help prognostic risk stratification in acute ICH patients with initial clinical information. Furthermore, the uICH score may help clinicians to triage patients in resource-limited settings.

Our study had several strengths: Firstly, we have established two new ICH scores, which both exhibited better performance in terms of predicting poor functional outcomes, compared with the oICH score. Our findings imply that the oICH score may not be an optimal method for risk stratification in uICH patients. Secondly, we found that a hematoma comprising two or more NCCT signs may increase the probability of predicting an unfavorable prognosis of ICH. Thus, incorporating blend sign, black hole sign, and island sign into the oICH score could significantly improve the accuracy for predicting poor functional outcomes and may provide additional prognostic information for clinical decision-making in patients with uICH.

Our study had several limitations that should be considered when interpreting its results. First, we did not evaluate the follow-up GCS score in this observational study. Thus, we could not determine follow-up oICH scores to calculate the risk of stabilized hematoma volume. Although we developed the dICH score to simulate a stable hematoma for the prediction of poor outcomes, follow-up oICH scores are important considerations in future research. Second, this was a single-center study with a relatively small sample, which may require large-scale multicenter studies to replicate our results in the future.

## Conclusions

The dICH score and the uICH score were useful clinical assessment tools that could be used to discriminate poor outcomes and provide guidance in clinical decision-making concerning patients with acute ICH.

## Data Availability Statement

The original contributions presented in the study are included in the article/Supplementary Material, further inquiries can be directed to the corresponding author/s.

## Ethics Statement

The studies involving human participants were reviewed and approved by the Ethics Committee of the First Affiliated Hospital of Chongqing Medical University. The patients/participants provided their written informed consent to participate in this study.

## Author Contributions

W-SY, QL, and PX were responsible for the study concept and design and had full access to all of the data in the study. W-SY, Y-QS, XW, L-BZ, Q-JL, X-FX, Z-WZ, LD, X-NL, S-QZ, X-HL, QL, and PX did the acquisition, analysis, or interpretation of data. W-SY drafted the manuscript. QL and PX did the critical revision of the manuscript and were responsible for the administrative, technical, or material support. W-SY, Y-QS, and XW did the statistical analysis. QL obtained fundings. All authors contributed to the article and approved the submitted version.

## Conflict of Interest

The authors declare that the research was conducted in the absence of any commercial or financial relationships that could be construed as a potential conflict of interest.
