# Corrigendum: New Prediction Models of Functional Outcome in Acute Intracerebral Hemorrhage: The dICH Score and uICH Score

**DOI:** 10.3389/fneur.2022.970106

**Published:** 2022-07-13

**Authors:** Wen-Song Yang, Yi-Qing Shen, Xiao Wei, Li-Bo Zhao, Qing-Jun Liu, Xiong-Fei Xie, Zhi-Wei Zhang, Lan Deng, Xin-Ni Lv, Shu-Qiang Zhang, Xin-Hui Li, Qi Li, Peng Xie

**Affiliations:** ^1^Department of Neurology, The First Affiliated Hospital of Chongqing Medical University, Chongqing, China; ^2^National Health Commission Key Laboratory of Diagnosis and Treatment on Brain Functional Diseases, The First Affiliated Hospital of Chongqing Medical University, Chongqing, China; ^3^Department of Traditional Chinese Medicine, Chongqing Medical and Pharmaceutical College, Chongqing, China; ^4^Department of Neurology, Yongchuan Hospital of Chongqing Medical University, Chongqing, China; ^5^Chongqing Key Laboratory of Cerebrovascular Disease Research, Yongchuan Hospital of Chongqing Medical University, Chongqing, China; ^6^Department of Radiology, The First Affiliated Hospital of Chongqing Medical University, Chongqing, China

**Keywords:** intracerebral hemorrhage, ICH score, hematoma expansion, outcome, NCCT marker, computed tomography

In the published article, there was an error. There were typographic mistakes for the numbers of patients. A correction has been made and the results section of the abstract now reads:

**Results:** There were 310 patients in this study which included 72 patients (23.2%) with hematoma expansion and 58 patients (18.7%) with IVH growth. Of 31 patients with two or more non-contrast computed tomography markers, 61.3% died, and 96.8% had poor outcomes at 90 days. After adjustment for potential confounding variables, we found that age, baseline Glasgow Coma Scale score, presence of IVH on initial CT, baseline ICH volume, infratentorial hemorrhage, hematoma expansion, IVH growth, blend sign, black hole sign, and island sign could independently predict poor outcomes in multivariate analysis. In comparison with the oICH score, the dICH score and uICH score exhibited better performance in the prediction of poor functional outcomes.

The authors apologize for this error and state that this does not change the scientific conclusions of the article in any way. The original article has been updated.

## Publisher's Note

All claims expressed in this article are solely those of the authors and do not necessarily represent those of their affiliated organizations, or those of the publisher, the editors and the reviewers. Any product that may be evaluated in this article, or claim that may be made by its manufacturer, is not guaranteed or endorsed by the publisher.

