# Supplementary material for: New Prediction Models of Functional Outcome in Acute Intracerebral Hemorrhage: The dICH Score and uICH Score
Source: Front Neurol. 2021 May 5;12:655800. doi: 10.3389/fneur.2021.655800 (PMC8131837; doi:10.3389/fneur.2021.655800)

**SUPPLEMENTARY TABLE 1 The oICH score, dICH score, and uICH score associated with poor outcome**

| **Outcome points** | Se., % | Sp., % | AUC | Optimal points | Youden Index |
| --- | --- | --- | --- | --- | --- |
| **30-day mortality** | | | | | |
| oICH score | 66.7 | 80.1 | 0.82(0.76-0.89) | 2 | 0.468 |
| uICH score | 87.2 | 69.7 | 0.86(0.80-0.91) | 2 | 0.569 |
| dICH score | 92.3 | 69.0 | 0.89(0.84-0.94) | 2 | 0.613 |
| **90-day mortality** | | | | | |
| oICH score | 62.7 | 82.9 | 0.82(0.76-0.88) | 2 | 0.456 |
| uICH score | 84.7 | 73.7 | 0.86(0.81-0.92) | 2 | 0.584 |
| dICH score | 88.1 | 72.9 | 0.88(0.83-0.93) | 2 | 0.610 |
| **Poor outcome** | | | | | |
| oICH score | 85.0 | 56.8 | 0.79(0.74-0.84) | 1 | 0.418 |
| uICH score | 71.7 | 84.2 | 0.85(0.80-0.89) | 2 | 0.559 |
| dICH score | 75.0 | 84.2 | 0.86(0.81-0.90) | 2 | 0.592 |

ICH indicates intracerebral hemorrhage; UNCT, ultraearly noncontrast computed tomography; Se., Sensitivity; Sp., Specificity; oICH, original intracerebral hemorrhage; dICH, dynamic intracerebral hemorrhage; uICH, ultra-early intracerebral hemorrhage.

**Figure Legend**

**SUPPLEMENTARY FIGURE 1 Cohort Selection Flowchart.** ICH indicates intracerebral hemorrhage; CT, computed tomography; IVH, intraventricular hemorrhage.


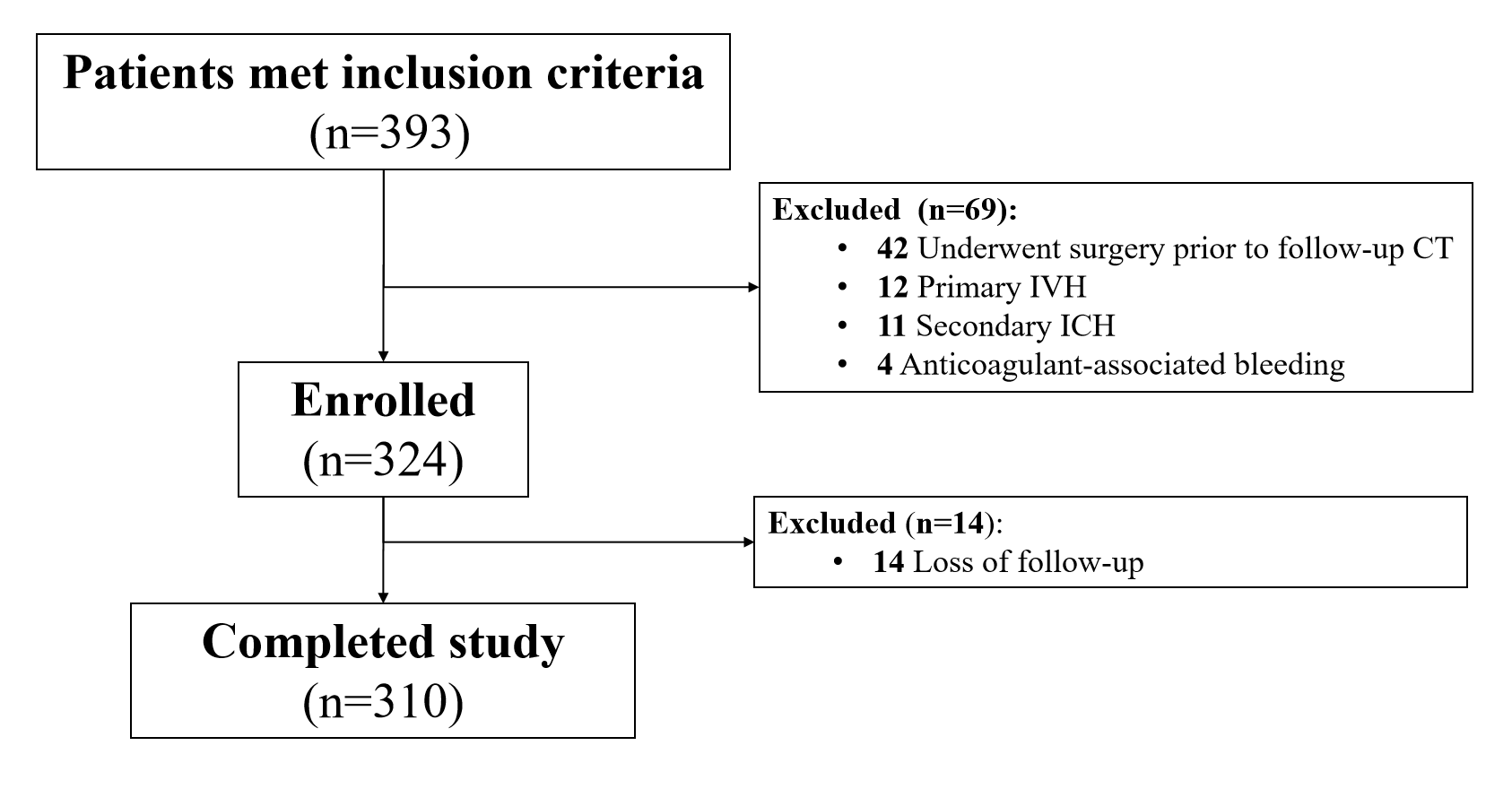


**SUPPLEMENTARY FIGURE 2 illustration of the segmentation of parenchymal hematoma and ventricular hematoma.** A, A hematoma at the parenchymal tissue. B, Building the mask with predefined values 44 to 99 hounsfield units (green and yellow mask) and confirming the hematoma with a region growing (yellow mask). C, Drawing the outline of the hematoma automatically (blue line). D, A hematoma presented in both ventricles and parenchyma. E, Dividing the connected hematoma into parenchymal hematoma (yellow mask) and ventricular hematoma (blue mask), respectively. F, Drawing the outline of the parenchymal hematoma (blue line) and ventricular hematoma (purple line), respectively.


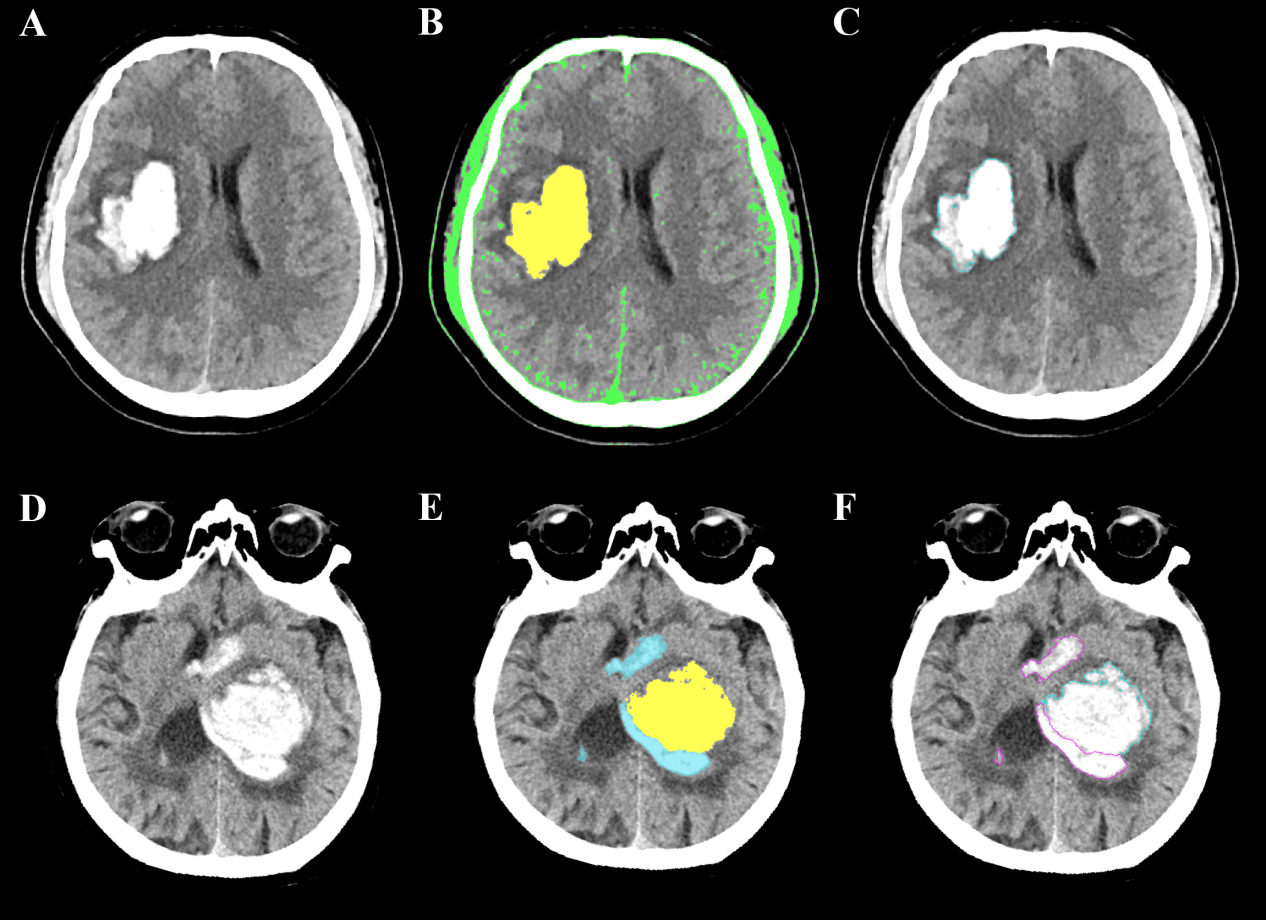

Supplement: Supplementary file 1 [file Data_Sheet_1.docx]
